# 2-Hy­droxy-6-isopropyl-3-methyl­benzoic acid

**DOI:** 10.1107/S1600536811011998

**Published:** 2011-04-07

**Authors:** Richard Betz, Thomas Gerber, Henk Schalekamp

**Affiliations:** a Nelson Mandela Metropolitan University, Summerstrand Campus, Department of Chemistry, University Way, Summerstrand, PO Box 77000, Port Elizabeth, 6031, South Africa

## Abstract

The title compound, C_11_H_14_O_3_, is a multiple-substituted derivative of benzoic acid. Intra­cyclic C—C—C angles span a range of 117.16 (19)–122.32 (19)°. Apart from intra­molecular hydrogen bonds between hydroxyl and carboxyl groups, inter­molecular hydrogen bonds are present in the crystal structure, the latter ones giving rise to centrosymmetric carb­oxy­lic acid dimers.

## Related literature

For the X-ray crystal structure of benzoic acid, see: Bruno & Randaccio (1980 ▶). For the crystal structure of benzoic acid applying neutron radiation, see: Wilson *et al.* (1996 ▶). For the crystal structure of *meta*-methyl­benzoic acid (without three-dimensional coordinates), see: Ellas & García-Blanco (1963 ▶). For a recent crystal structure analysis of salicylic acid, see: Munshi & Guru Row (2006 ▶). For graph-set analysis of hydrogen bonds, see: Etter *et al.* (1990 ▶); Bernstein *et al.* (1995 ▶).
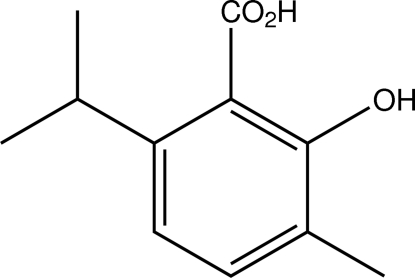


## Experimental

### 

#### Crystal data

C_11_H_14_O_3_
*M* *_r_* = 194.22
Orthorhombic, 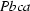

*a* = 16.8864 (17) Å
*b* = 6.6653 (7) Å
*c* = 18.238 (2) Å
*V* = 2052.7 (4) Å^3^
*Z* = 8
Mo *K*α radiation
μ = 0.09 mm^−1^
*T* = 200 K
0.51 × 0.16 × 0.08 mm

#### Data collection

Bruker APEXII CCD diffractometer
10325 measured reflections
2546 independent reflections
1365 reflections with *I* > 2σ(*I*)
*R*_int_ = 0.073

#### Refinement

*R*[*F* ^2^ > 2σ(*F* ^2^)] = 0.054
*wR*(*F* ^2^) = 0.154
*S* = 0.99
2546 reflections
132 parameters
H-atom parameters constrained
Δρ_max_ = 0.30 e Å^−3^
Δρ_min_ = −0.24 e Å^−3^

### 

Data collection: *APEX2* (Bruker, 2010 ▶); cell refinement: *SAINT* (Bruker, 2010 ▶); data reduction: *SAINT*; program(s) used to solve structure: *SHELXS97* (Sheldrick, 2008 ▶); program(s) used to refine structure: *SHELXL97* (Sheldrick, 2008 ▶); molecular graphics: *ORTEPIII* (Farrugia, 1997 ▶) and *Mercury* (Macrae *et al.*, 2006 ▶); software used to prepare material for publication: *SHELXL97* and *PLATON* (Spek, 2009 ▶).

## Supplementary Material

Crystal structure: contains datablocks I, global. DOI: 10.1107/S1600536811011998/bh2346sup1.cif

Structure factors: contains datablocks I. DOI: 10.1107/S1600536811011998/bh2346Isup2.hkl

Additional supplementary materials:  crystallographic information; 3D view; checkCIF report

## Figures and Tables

**Table 1:** Hydrogen-bond geometry (Å, °)

*D*—H⋯*A*	*D*—H	H⋯*A*	*D*⋯*A*	*D*—H⋯*A*
O3—H3⋯O2	0.84	1.77	2.5171 (19)	146
O1—H1⋯O2^i^	0.84	1.81	2.6475 (19)	174

Symmetry code: (i) 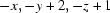.

**Fig. 1.**
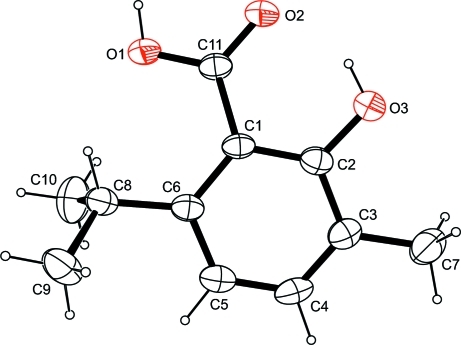
The molecular structure of the title compound, with anisotropic displacement ellipsoids drawn at 50% probability level.

**Fig. 2.**
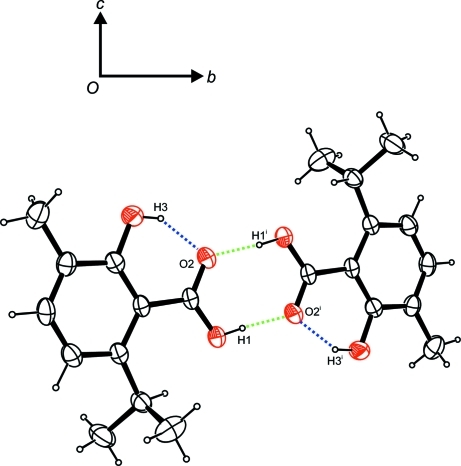
Intramolecular (blue dashed lines) and intermolecular (green dashed lines) contacts, viewed along [-1 0 0]. Symmetry operator: i -x, -y+2, -z+1.

**Fig. 3.**
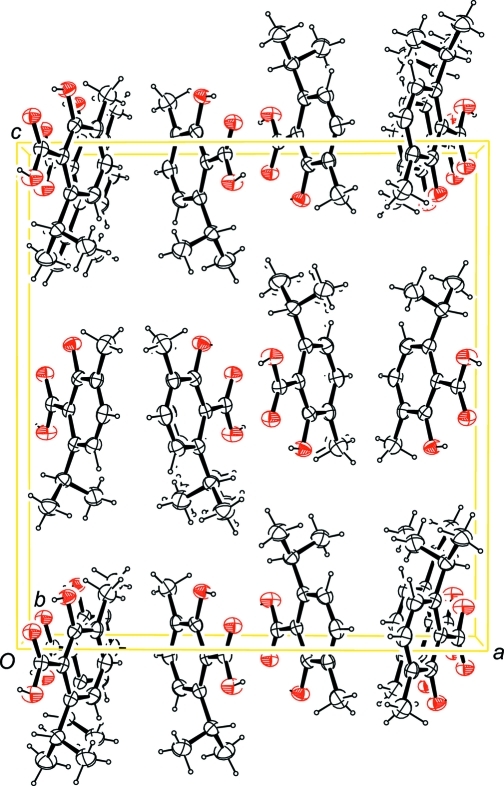
Molecular packing of the title compound, viewed along [0 1 0] (anisotropic displacement ellipsoids drawn at 50% probability level).

## supplementary crystallographic information

### Comment

Benzoic acid has found widespread use as a ligand in coordination chemistry for a variety of transition metals and elements from the *s*- and *p*-block of the periodic system of the elements. It can act as a neutral or – upon deprotonation – an anionic ligand and serve as mono- or bidentate ligand. By varying the substituents on the phenyl moiety, the acidity of the carboxylic acid group can be fine-tuned. Particular interest rests in benzoic acid derivatives showing an asymmetric pattern of substituents on the aromatic moiety due to different possible orientations of the ligand in coordination compounds and the possible formation of stereoisomeric products. At the beginning of a comprehensive study aimed at rationalizing the coordination behaviour of various benzoic acid derivatives towards a number of transition metals in dependence of the pH value of the reaction batches it seemed interesting to determine the crystal structure of the title compound to enable comparative studies. The crystal structure of unsubstituted benzoic acid (Bruno & Randaccio, 1980; Wilson *et al.*, 1996) as well as the crystal structures of *meta*-methylbenzoic acid (Ellas & García-Blanco, 1963; three-dimensional coordinates not deposited) and salicylic acid (Munshi & Guru Row, 2006) are apparent in the literature.

C—C—C angles within the carbocyclic ring span a range of 117–122°. The two smallest angles are found on the C atoms bearing the alkyl substituents while the two biggest angles are found on the C atom bearing the hydroxyl group and the C atom in *para*-position to the one bonded to the alcoholic hydroxyl group.

While the isopropyl group is tilted significantly in relation to the benzene moiety, the carboxylic acid group is nearly in plane with the carbocycle. The least-squares planes defined by the C atoms of the isopropyl group as well as the C atoms of the benzene group, respectively, enclose an angle of 74.17 (9)°, while the least-squares planes defined by the atoms of the carboxylic acid group on the one hand and the carbon atoms of the aromatic moiety on the other hand intersect at an angle of only 7.07 (28)° (Fig. 1).

In the crystal structure, intra- as well as intermolecular hydrogen bonds can be observed. The intramolecular hydrogen bonds are formed by the H atom of the alcoholic hydroxyl group as the donor and the carbonylic O atom of the carboxylic acid group as the acceptor. The intermolecular hydrogen bonds are apparent between carboxylic acid groups connecting two neighbouring molecules to centrosymmetric dimers. In terms of graph-set analysis (Etter *et al.*, 1990; Bernstein *et al.*, 1995), the descriptor for the intramolecular motif is *S*^1^_1_(6) on the unitary level while the intermolecular hydrogen bonds necessitate a *R*^2^_2_(8) descriptor on the same level (Fig. 2). The shortest *C*_g_···*C*_g_ distance for benzene rings in the crystal was measured at 4.9918 (13) Å.

The packing of the title compound is shown in Figure 3.

### Experimental

The compound was obtained commercially (Aldrich). Crystals suitable for the X-ray diffraction study were taken directly from the provided product.

### Refinement

Carbon-bound H-atoms were placed in calculated positions (C—H 0.95 Å for aromatic C atoms, C—H 1.00 Å for the methine group and C—H 0.98 Å for methyl groups) and were included in the refinement in the riding model approximation, with *U*_iso_(H) set to 1.2*U*_eq_(C) or 1.5*U*_eq_(C). Oxygen-bound H-atoms were placed in calculated positions with O—H = 0.84 Å and *U*_iso_(H) = 1.5*U*_eq_(O). The methyl groups were allowed to rotate with a fixed angle around the C—C bonds to best fit the experimental electron density [AFIX 137 in the *SHELX* program suite (Sheldrick, 2008)]. The H atom of the carboxylic acid group as well as the hydroxyl group were allowed to rotate with a fixed angle around the C—O bonds to best fit the experimental electron density [AFIX 147 in the *SHELX* program suite (Sheldrick, 2008)].

### Crystal data

**Table:**

C_11_H_14_O_3_	*F*(000) = 832
*M**_r_* = 194.22	*D*_x_ = 1.257 Mg m^−^^3^
Orthorhombic, *P**b**c**a*	Mo *K*α radiation, λ = 0.71073 Å
Hall symbol: -P 2ac 2ab	Cell parameters from 1806 reflections
*a* = 16.8864 (17) Å	θ = 3.3–26.5°
*b* = 6.6653 (7) Å	µ = 0.09 mm^−^^1^
*c* = 18.238 (2) Å	*T* = 200 K
*V* = 2052.7 (4) Å^3^	Rod, colourless
*Z* = 8	0.51 × 0.16 × 0.08 mm

### Data collection

**Table:**

Bruker APEXII CCD diffractometer	1365 reflections with *I* > 2σ(*I*)
Radiation source: fine-focus sealed tube	*R*_int_ = 0.073
graphite	θ_max_ = 28.3°, θ_min_ = 3.3°
φ and ω scans	*h* = −18→22
10325 measured reflections	*k* = −8→8
2546 independent reflections	*l* = −16→24

### Refinement

**Table:**

Refinement on *F*^2^	Primary atom site location: structure-invariant direct methods
Least-squares matrix: full	Secondary atom site location: difference Fourier map
*R*[*F*^2^ > 2σ(*F*^2^)] = 0.054	Hydrogen site location: inferred from neighbouring sites
*wR*(*F*^2^) = 0.154	H-atom parameters constrained
*S* = 0.99	*w* = 1/[σ^2^(*F*_o_^2^) + (0.076*P*)^2^] where *P* = (*F*_o_^2^ + 2*F*_c_^2^)/3
2546 reflections	(Δ/σ)_max_ < 0.001
132 parameters	Δρ_max_ = 0.30 e Å^−^^3^
0 restraints	Δρ_min_ = −0.24 e Å^−^^3^
0 constraints	

### Fractional atomic coordinates and isotropic or equivalent isotropic displacement parameters (Å^2^)

**Table:**

	*x*	*y*	*z*	*U*_iso_*/*U*_eq_	
O1	0.04216 (9)	0.85849 (19)	0.42308 (8)	0.0409 (4)	
H1	0.0189	0.9643	0.4358	0.061*	
O2	0.03999 (9)	0.8162 (2)	0.54297 (8)	0.0357 (4)	
O3	0.10701 (9)	0.5223 (2)	0.60346 (7)	0.0386 (4)	
H3	0.0830	0.6324	0.6010	0.058*	
C1	0.09835 (11)	0.5574 (3)	0.47072 (11)	0.0262 (4)	
C2	0.12250 (11)	0.4550 (3)	0.53481 (11)	0.0297 (5)	
C3	0.16463 (12)	0.2740 (3)	0.53193 (11)	0.0325 (5)	
C4	0.18243 (12)	0.1994 (3)	0.46369 (12)	0.0369 (5)	
H4	0.2128	0.0799	0.4601	0.044*	
C5	0.15747 (12)	0.2931 (3)	0.40016 (12)	0.0364 (5)	
H5	0.1703	0.2341	0.3543	0.044*	
C6	0.11437 (11)	0.4699 (3)	0.40105 (11)	0.0286 (5)	
C7	0.18707 (14)	0.1685 (3)	0.60150 (13)	0.0463 (6)	
H71	0.2190	0.0499	0.5898	0.069*	
H72	0.1390	0.1269	0.6275	0.069*	
H73	0.2178	0.2595	0.6326	0.069*	
C8	0.08638 (12)	0.5538 (3)	0.32753 (12)	0.0345 (5)	
H8	0.0399	0.6429	0.3373	0.041*	
C9	0.05933 (15)	0.3885 (4)	0.27477 (14)	0.0525 (7)	
H91	0.1053	0.3094	0.2592	0.079*	
H92	0.0342	0.4494	0.2318	0.079*	
H93	0.0212	0.3009	0.2996	0.079*	
C10	0.15119 (15)	0.6819 (4)	0.29282 (14)	0.0563 (7)	
H101	0.1669	0.7880	0.3271	0.084*	
H102	0.1312	0.7426	0.2475	0.084*	
H103	0.1971	0.5975	0.2816	0.084*	
C11	0.05805 (11)	0.7512 (3)	0.48107 (11)	0.0282 (5)	

### Atomic displacement parameters (Å^2^)

**Table:**

	*U*^11^	*U*^22^	*U*^33^	*U*^12^	*U*^13^	*U*^23^
O1	0.0625 (11)	0.0252 (7)	0.0349 (9)	0.0137 (7)	0.0030 (8)	−0.0027 (7)
O2	0.0455 (9)	0.0275 (7)	0.0342 (8)	0.0079 (6)	0.0033 (7)	−0.0065 (6)
O3	0.0473 (9)	0.0373 (8)	0.0313 (9)	0.0094 (7)	0.0050 (7)	−0.0009 (7)
C1	0.0232 (9)	0.0204 (8)	0.0350 (12)	−0.0017 (7)	0.0039 (8)	−0.0037 (8)
C2	0.0287 (10)	0.0272 (10)	0.0332 (12)	−0.0025 (8)	0.0050 (9)	−0.0038 (9)
C3	0.0281 (10)	0.0278 (10)	0.0416 (13)	−0.0012 (8)	0.0038 (9)	0.0031 (9)
C4	0.0332 (11)	0.0247 (10)	0.0530 (15)	0.0069 (8)	0.0056 (10)	−0.0016 (10)
C5	0.0380 (12)	0.0321 (10)	0.0392 (13)	0.0042 (9)	0.0056 (10)	−0.0078 (10)
C6	0.0279 (10)	0.0244 (9)	0.0334 (12)	−0.0016 (8)	0.0032 (9)	−0.0043 (9)
C7	0.0473 (13)	0.0382 (11)	0.0534 (16)	0.0075 (10)	0.0047 (12)	0.0107 (11)
C8	0.0371 (11)	0.0332 (10)	0.0330 (12)	0.0053 (9)	−0.0002 (9)	−0.0070 (9)
C9	0.0639 (16)	0.0539 (15)	0.0399 (15)	0.0033 (12)	−0.0049 (12)	−0.0178 (12)
C10	0.0536 (15)	0.0668 (16)	0.0485 (16)	−0.0077 (13)	−0.0021 (12)	0.0183 (13)
C11	0.0290 (10)	0.0202 (9)	0.0355 (12)	−0.0020 (8)	0.0003 (9)	−0.0048 (8)

### Geometric parameters (Å, °)

**Table:**

O1—C11	1.305 (2)	C5—H5	0.9500
O1—H1	0.8400	C6—C8	1.528 (3)
O2—C11	1.247 (2)	C7—H71	0.9800
O3—C2	1.355 (2)	C7—H72	0.9800
O3—H3	0.8400	C7—H73	0.9800
C1—C2	1.414 (3)	C8—C10	1.526 (3)
C1—C6	1.424 (3)	C8—C9	1.533 (3)
C1—C11	1.472 (3)	C8—H8	1.0000
C2—C3	1.402 (3)	C9—H91	0.9800
C3—C4	1.373 (3)	C9—H92	0.9800
C3—C7	1.499 (3)	C9—H93	0.9800
C4—C5	1.382 (3)	C10—H101	0.9800
C4—H4	0.9500	C10—H102	0.9800
C5—C6	1.385 (3)	C10—H103	0.9800
			
C11—O1—H1	109.5	H71—C7—H73	109.5
C2—O3—H3	109.5	H72—C7—H73	109.5
C2—C1—C6	119.03 (17)	C10—C8—C6	110.31 (18)
C2—C1—C11	116.80 (17)	C10—C8—C9	110.84 (19)
C6—C1—C11	124.16 (18)	C6—C8—C9	112.35 (18)
O3—C2—C3	114.68 (18)	C10—C8—H8	107.7
O3—C2—C1	123.25 (17)	C6—C8—H8	107.7
C3—C2—C1	122.07 (18)	C9—C8—H8	107.7
C4—C3—C2	117.16 (19)	C8—C9—H91	109.5
C4—C3—C7	122.81 (19)	C8—C9—H92	109.5
C2—C3—C7	120.02 (19)	H91—C9—H92	109.5
C3—C4—C5	121.99 (19)	C8—C9—H93	109.5
C3—C4—H4	119.0	H91—C9—H93	109.5
C5—C4—H4	119.0	H92—C9—H93	109.5
C4—C5—C6	122.32 (19)	C8—C10—H101	109.5
C4—C5—H5	118.8	C8—C10—H102	109.5
C6—C5—H5	118.8	H101—C10—H102	109.5
C5—C6—C1	117.30 (18)	C8—C10—H103	109.5
C5—C6—C8	117.62 (17)	H101—C10—H103	109.5
C1—C6—C8	125.07 (17)	H102—C10—H103	109.5
C3—C7—H71	109.5	O2—C11—O1	119.55 (17)
C3—C7—H72	109.5	O2—C11—C1	122.27 (18)
H71—C7—H72	109.5	O1—C11—C1	118.17 (17)
C3—C7—H73	109.5		
			
C6—C1—C2—O3	−177.21 (18)	C2—C1—C6—C5	−3.8 (3)
C11—C1—C2—O3	3.1 (3)	C11—C1—C6—C5	175.81 (17)
C6—C1—C2—C3	2.7 (3)	C2—C1—C6—C8	174.92 (18)
C11—C1—C2—C3	−176.96 (17)	C11—C1—C6—C8	−5.4 (3)
O3—C2—C3—C4	−179.57 (18)	C5—C6—C8—C10	−85.4 (2)
C1—C2—C3—C4	0.5 (3)	C1—C6—C8—C10	95.8 (2)
O3—C2—C3—C7	1.5 (3)	C5—C6—C8—C9	38.8 (3)
C1—C2—C3—C7	−178.43 (18)	C1—C6—C8—C9	−140.0 (2)
C2—C3—C4—C5	−2.6 (3)	C2—C1—C11—O2	−5.6 (3)
C7—C3—C4—C5	176.35 (19)	C6—C1—C11—O2	174.72 (18)
C3—C4—C5—C6	1.3 (3)	C2—C1—C11—O1	173.29 (17)
C4—C5—C6—C1	1.9 (3)	C6—C1—C11—O1	−6.4 (3)
C4—C5—C6—C8	−176.91 (19)		

### Hydrogen-bond geometry (Å, °)

**Table:**

*D*—H···*A*	*D*—H	H···*A*	*D*···*A*	*D*—H···*A*
O3—H3···O2	0.84	1.77	2.5171 (19)	146
O1—H1···O2^i^	0.84	1.81	2.6475 (19)	174

Symmetry codes: (i) −*x*, −*y*+2, −*z*+1.
